# Best Practice in the chemical characterisation of extracts used in pharmacological and toxicological research—The ConPhyMP—Guidelines^1^ ^2^

**DOI:** 10.3389/fphar.2022.953205

**Published:** 2022-09-13

**Authors:** Michael Heinrich, Banaz Jalil, Mona Abdel-Tawab, Javier Echeverria, Žarko Kulić, Lyndy J. McGaw, John M. Pezzuto, Olivier Potterat, Jia-Bo Wang

**Affiliations:** ^1^ Pharmacognosy and Phytotherapy, UCL School of Pharmacy, London, United Kingdom; ^2^ Central Laboratory of German Pharmacists, Eschborn, Germany/Institute of Pharmaceutical Chemistry, Johann-Wolfgang-Goethe University, Frankfurt, Germany; ^3^ Departamento de Ciencias del Ambiente, Facultad de Química y Biología, Universidad de Santiago de Chile, Santiago, Chile; ^4^ Preclinical Research and Development, Dr. Willmar Schwabe GmbH & Co. KG, Karlsruhe, Germany; ^5^ Phytomedicine Programme, Department of Paraclinical Sciences, Faculty of Veterinary Science, University of Pretoria, Pretoria, South Africa; ^6^ College of Pharmacy and Health Sciences, Western New England University, Springfield, MA, United States; ^7^ Division of Pharmaceutical Biology, University of Basel, Basel, Switzerland; ^8^ School of Traditional Chinese Medicine, Capital Medical University, Beijing, China

**Keywords:** Best practice, extract characterisation, medicinal plant, analytical methods, phytochemical analysis, HPTLC, HPLC

## Abstract

**Background:** Research on medicinal plants and extracts derived from them differs from studies performed with single compounds. Extracts obtained from plants, algae, fungi, lichens or animals pose some unique challenges: they are multicomponent mixtures of active, partially active and inactive substances, and the activity is often not exerted on a single target. Their composition varies depending on the method of preparation and the plant materials used. This complexity and variability impact the reproducibility and interpretation of pharmacological, toxicological and clinical research.

**Objectives:** This project develops best practice guidelines to ensure reproducibility and accurate interpretations of studies using medicinal plant extracts. The focus is on herbal extracts used in pharmacological, toxicological, and clinical/intervention research. Specifically, the consensus-based statement focuses on defining requirements for: 1) Describing the plant material/herbal substances, herbal extracts and herbal medicinal products used in these studies, and 2) Conducting and reporting the phytochemical analysis of the plant extracts used in these studies in a reproducible and transparent way.

**The process and methods:** We developed the guidelines through the following process: 1) The distinction between the three main types of extracts (extract types A, B, and C), initially conceptualised by the lead author (MH), led the development of the project as such; 2) A survey among researchers of medicinal plants to gather global perspectives, opportunities, and overarching challenges faced in characterising medicinal plant extracts under different laboratory infrastructures. The survey responses were central to developing the guidelines and were reviewed by the core group; 3) A core group of 9 experts met monthly to develop the guidelines through a Delphi process; and. 4) The final draft guidelines, endorsed by the core group, were also distributed for feedback and approval to an extended advisory group of 20 experts, including many journal editors.

**Outcome:** The primary outcome is the “Consensus statement on the Phytochemical Characterisation of Medicinal Plant extracts“ (ConPhyMP) which defines the best practice for reporting the starting plant materials and the chemical methods recommended for defining the chemical compositions of the plant extracts used in such studies. The checklist is intended to be an orientation for authors in medicinal plant research as well as peer reviewers and editors assessing such research for publication.

## 1 Introduction

### 1.1 What are the challenges with medicinal plant extracts?

Every year, thousands of studies evaluate the pharmacological effects (clinical) efficacy or the toxicity of medicinal plant extracts. These studies attest to the importance of herbal medicines. Researchers involved in these studies face unique challenges (Sticher, 2008; Kinghorn et al., 2011) due to the complexity and often poor characterisation of the test item as compared to defined single chemical entities in the classical drug development area. Medicinal plant extracts differ from their chemically defined pharmaceutical counterparts in that they are typically complex mixtures, where the identities and quantities of the active ingredients or marker compounds present are not fully known (Heinrich et al., 2017). In many countries, especially the fast developing economies, the vast majority of the population (about 80%) depends almost totally on natural remedies for primary health care needs. A comprehensive global summation has recently been published (World Health Organisation, 2019), and guidance has been provided for the assessment of safety and efficacy (World Health Organisation, 2000), with both studies published under the auspices of the World Health Organisation.

As described in the oft-cited synopses by Newman and Cragg (2020), a considerable number of contemporary drugs are natural products, or bear a relationship to natural products. The scientific community has grown very adept at dealing with these different drug entities. For example, sophisticated methods of isolation and structure elucidation can be applied, purity can be precisely determined and detailed mechanistic evaluations can be performed. Additionally, factors such as metabolism and distribution (pharmacokinetics) are determined, structure-activity relationships are studied, as are toxicity and efficacy. Importantly, all of the tenets of the ‘scientific method’ apply. In particular, it is essential that work reported in the literature can be replicated by others skilled in the art, and new hypotheses can be developed and tested based on foundational knowledge grounded in fact. While not underestimating the ingenuity and scientific intuition required for the discovery of natural product based medicine, for the most part, working with a pure chemical entity is arguably relatively straightforward. Continuity is assured, and progress can be realized, one logical step after another. However, this is generally not the case when pharmacological research is performed with poorly defined bioactive herbal preparations. As an attempt to help rectify this situation, we have recently provided some perspective on what may be viewed as ‘best practice’ in early stage phytopharmacological research (Heinrich et al., 2020). At the far end of the spectrum, late-stage translation for the conduct of actual clinical trials, a comprehensive treatise on how to manage phytopharmacological preparations, has been presented by Sorkin et al. (2020).

It is generally professed that several components may contribute to a synergistic response, and sometimes the therapeutic response is related to a traditional system of medicine including other elements beyond the drug administration. Furthermore, it is often presumed that safety and efficacy cannot be fully retained or replicated by distilling the substances contained in the natural remedy down to one or more pure chemical entities due to the complex interplay (e.g., pharmacokinetics and pharmacodynamics) between the different constituents of a complex mixture. Otherwise, a well-defined formulation could be developed and applied by the medical profession throughout the world. But is it feasible to produce refined material (i.e., a conglomeration of pure compounds) that could be utilized in a reproducible manner similar to “chemical entities”?

One of the lead authors has recently considered the concept of synergy (or additivity) in the context of combining multiple pure cancer chemopreventive agents (Pezzuto and Vang, 2020). In this case, the chemical structure and purity are known from the outset, and each chemical entity is known to mediate some type of biological response believed to be pertinent. Based on this information, at least in principle, an approach can be designed to systematically investigate synergism. However, such an undertaking is not trivial.

As an example, consider an approach for evaluating four bioactive compounds: **A**, **B**, **C**, and **D** (Pezzuto and Vang, 2020). Assuming an *in vitro* assay is available that is reflective of a germane mechanism, the initial analyses would involve six unique combinations (**A** + **B**, **A** + **C**, **A** + **D**, **B** + **C**, **B** + **D**, and **C** + **D**). Using a method such as isobolographic analysis, it could be established if these sets of agents function in an additive or synergistic manner. Once this is accomplished, these sets of the compounds could be evaluated with a third agent. This results in up to 12 additional sets [(**A** + **B**)+**C** (**A** + **B**)+**D** (**A** + **C**)+**B** (**A** + **C**)+**D** (**A** + **D**)+**B** (**A** + **D**)+**C** (**B** + **C**)+**A** (**B** + **C**)+**D** (**B** + **D**)+**A** (**B** + **D**)+**C** (**C** + **D**)+**A**, and (**C** + **D**)+**B**], when it is assumed that all the combinations are not equivalent [e.g., (**A** + **B**)+**C** ≠ (**A** + **C**)+**B**]. Again, the tests could be performed to investigate additive or synergistic responses. Finally, the fourth agent could be added to the combination and tested. For this, there are four unique combinations [(**A** + **B** + **C**) +**D** (**A** + **B** + **D**) +**C** (**A** + **C** + **D**) +**B**, and (**B** + **C** + **D**) +**A**]. Since tedious dose-response curves would need to be performed with each of the combinations listed above, the gargantuan magnitude of such an effort is apparent. And this assumes the availability of a single *in vitro* assay that is truly reflective of an *in vivo* response, which is not a likely reality.

Nonetheless, to some extent, these same principles could be applied to multicomponent traditional medicines. On the one hand, it is encouraging that the combination index as described by Chou (2006) can be applied for preparations that do not need to be pure chemical entities. Thus, systematic evaluation of combined mixtures of multiple components could be undertaken. However, this approach is generally not applicable, since a specific and meaningful mechanism of action is unknown. The situation is further complicated by the potential of the complete preparation to mediate an immunological response concomitantly with other target-based mechanisms. Thus, the action of a polyprescription is perceived to be too complex to realistically isolate active principles and admix the resulting substances to yield the same response as the starting plant material. In summary, component analysis of a traditional medicine polyprescription preparation is simply not practicable.

An alternative would be to consider biological/pharmacological standardization of a traditional or herbal medicine in the context of biological units. From the Plant Kingdom, a historical example of this approach is a procedure designed to quantify the potency of (chemically uncharacterised) digitalis glycosides by assessing emetic effects with pigeons (Burn, 1930). Perhaps more widely known, penicillin is a prototypical example of this concept. There is an extensive history associated with the development of penicillin, but essentially, original penicillin was an ill-defined mixture of active compounds. Since the potency varied from batch-to-batch, antibiotic activity was determined, and preparations could be standardized based on units of biological activity. In 1959, it was determined that one unit of penicillin is equivalent to the antimicrobial activity produced by 0.59 µg of pure penicillin (Humphrey et al., 1959). Thus, it is not necessary to standardize penicillin based on antimicrobial activity, but still, by convention in the United States, the concentration of penicillin is often expressed in units. If such a situation existed for the standardization of unrefined plant material, i.e., accurate definition of the biological activity of a plant extract in terms of units, the establishment of such a convention would provide experimental continuity. Clinicians and scientists could all work with different preparations known to yield the same biological response based on units of activity. However, based on the complexities described above, this too is not feasible.

In turn, this begs the question of what scientific criteria should be deemed acceptable for conducting studies with natural product based remedies that are worthy of publication in the scientific literature. It contradicts basic principles of good scientific practice when published research cannot be reproduced by others. This would represent little more than an anecdote. Perhaps even worse, the results often are misleading for those who attempt to build on non-reproducible, published data. Essentially, results obtained with ill-gotten starting plant materials and their publication could well be viewed as the antithesis of the “scientific method”.

### 1.2 What is needed?

This paper sets out to define what is considered as “best practice” to enable the *reproducibility of research on the pharmacological and biological activity of plant extracts, i.e., complex mixtures of natural products (metabolites) and the reporting of such information*. Medicinal plant research is a thriving field of research. It covers a field of scientific investigation including medicinal plant research, ethnopharmacology, phytomedicine, phytotherapy research and natural health products for use both in humans and animals. Here we refer to all of these aspects inclusively as *medicinal plant research*, recognising the wide scope and complexity of the field.

It is difficult for one person to possess high-level expertise in all of these specialised fields—pharmacology or toxicology or clinical research in one area of medicine (e.g., gastrointestinal diseases) combined with expertise in analytical chemistry [(phyto)chemical analysis], plant sciences and also an understanding of the therapeutic or other uses of these plants (i.e., ethnobotany). It is a prime example of a multidisciplinary field, and the aim needs to be to integrate these fields into a transdisciplinary approach. As scientists, we venture into areas new to us, or at least areas in which we have received little formal training. This paper provides a basic overview of analytical strategies recommended to be used to characterise extracts used in such pharmacological research, to an extent, which facilitates comparability and/or reproducibility of the data.

Triggered by a critique that pharmacological research lacks reproducibility, Ioannidis highlighted the numerous risks and biases in pharmacological research. This includes scientific factors like when effect sizes are smaller, using inappropriate statistical methods to account for the number of tested relationships and a limited preselection of these parameters, as well as “greater flexibility in designs, definitions, outcomes, and analytical modes” (Ioannidis, 2005). Importantly, financial, reputational and other interests and prejudices are additional sources of bias in the interpretation of the results. This has reinforced the drive for developing standards, often ones which are journal specific, for example in the field of pharmacology (Curtis et al., 2018).

However, there is a noteworthy absence of a focus on the drug substance or medicine as such and on the primary material it is derived from. This is a particular challenge when complex mixtures are used, as is the case with medicinal plant extracts. In this regard, chemical analytical profiles of herbal extracts may also be considered as a major topic when considering pharmacological, toxicological and clinical/intervention studies of food supplements and herbal medicines. There are, of course, numerous biological, chemical and conceptual factors which influence the composition of an extract used, including:

- The growing conditions and initial processing/storage of the plant material (botanical material) and the resulting botanical drug.
- Time, e.g., collection date, development stage, harvesting protocols and damage by/response to pests and diseases.
- The different forms of processing (extraction) of these materials.
- Their preparation and mode of application when used for *in vitro* or *in vivo* experiments.
- Contamination with exogenous substances like pesticides and heavy metals.

Difficulties in profiling medicinal plant extracts may be attributed to the complexity of herbal extracts, and the variability of the concentrations of beneficial and toxic natural compounds due to seasonal/geographical differences in the plant material, as well as major or minor differences in the applied production processes.

Obviously, the choice of extraction solvents and the protocol used determine which compounds can be extracted. Whatever solvent one uses, there will always be some poorly extracted compounds and significant changes in activity may not be apparent when testing the effect of variables. Having reached the compounds’ maximum solubility is the most likely cause and, therefore, no changes can be observed in the next step, namely the quantitative analysis.

The ideal extraction solvent will depend on the type of plant material being extracted, the intended methods used to prepare the extract, and the research question. The choice of extraction solvent needs to be considered carefully and justified based on the experimental data and practical considerations (i.e., whether the extract is intended for human use).

In order for the research outcome to be generalizable, it is important to have some understanding of how representative is the plant extract or product used in these studies. This would involve how the natural variation of the chemical composition in the plant material (see above) is controlled. Testing the stability of the plant extracts over the study duration is an important factor to be considered.

Overall, *two herbal extracts can differ significantly in their composition, although they are prepared from the same plant species and based on a superficially similar protocol.*

Preparations of plants with canonical use as medicinal products are commonly included in national and/or regional pharmacopoeial monographs, and, thus, have a regulated status (licensed, listed or registered medicines). The monographs often specify many parameters of the preparation relating to the specific composition and quality of herbal medicines. Examples include the drug-to-extract-ratio (DER), drug-solvent-ratio (DSR), extraction solvent, chemical marker concentrations and daily doses. The aim of these monographs is to enable the extrapolation of general safety and/or efficacy data from the long-term use of similarly prepared botanical preparations to a given preparation. Yet, even if a preparation fulfills all specifications of the respective monograph, a simplified toxicological assessment (e.g., Ames test) and quality specifications are still required for registration of an herbal medical product in Europe. This is attributed to the possible differences in composition of herbal extracts, which are generally poorly defined via the specifications described in a pharmacopoeia. Often, some major parameters are not addressed in the monographs, like duration and temperature of the extraction process. Therefore, compliance only to pharmacopoeial standards is likely to be insufficient to characterize an extract.

To ensure the reproducibility and the wider interpretation of studies using plant extracts, selecting, characterising and assessing the continued quality and consistency of the plant material is an important step. The ideal characteristics of a medicinal plant extract or product used for research studies are:

- It should be authenticated and characterised in terms of active ingredients/marker compounds, of sufficient quality and consistency, and
- It should be stable (Table 1).

**TABLE 1:** Characteristics of the ideal medicinal plant extract or product used in pharmacological, toxicological and clinical/intervention studies.

1	Authentic
2	Well characterised
	Active ingredients known
	The chemical profile of the active ingredients/marker compounds is characterised qualitatively and quantitatively
3	Free of adulteration and contamination
4	Consistent
	Batch to batch variation is limited
5	Stable

In general, *pharmacopoeial standards and basic preparation protocols are not sufficient to fully characterize an extract; additional chemical characterization is needed*, due to the aforementioned reasons.

Over the past decades, there have been tremendous advances in the development of analytical methodologies. Today, there is a broad array of methods available, ranging from highly sophisticated and specialised (but also expensive) methods to ones which are robust and generic (and generally much more widely available and affordable). Such techniques include targeted and non-targeted chemical fingerprinting, like TLC/HPTLC, HPLC-UV, LC-MS, GC, GC-MS and NMR, to name only the most prominent^3^.

### 1.3 Why a stakeholder consultation and Delphi process?

It would be easy to define best practice criteria based on what one would want to do in an ideal research environment. However, there are numerous limitations to a best practice statement which is based on the views only of a small number of experts. Numerous methods are available and authors may select from these based on local availability and expertise. Many investigators have limited access to optimal methodology, which needs to be taken into consideration. Therefore, a stakeholder survey provides a basis for assessing what is feasible in various environments. Secondly, a Delphi process enabled us to define a consensus of best practice options. The authors of this paper contributed to the design of these guidelines. Further feedback was sought via social media and, moreover, the guidelines draft was also reviewed by a wider advisory group of 20 experts (the ConPhyMP panel).

## 2 Overarching aims and objectives

This project develops best practice guidelines on reporting the chemical composition of plant extracts to support the reproducibility of studies using medicinal plant extracts. The focus is on extracts used in pharmacological, toxicological and clinical research, aiming at ascertaining the reproducibility and interpretability of such studies. Specifically, the guidelines focus on defining the requirements needed for: 1) defining the plant material, herbal substances, herbal extracts and herbal medicinal products used in pharmacological, toxicological and clinical research, and 2) conducting and reporting the phytochemical analysis of the plant extracts used in these studies in a reproducible and transparent way. Initially, we aimed to understand the needs and expectations of researchers in the field. Therefore, we conducted a survey among scientists studying herbal extracts covering commonly used methods for chemical characterization.

## 3 The process and methods

### 3.1 Development and distribution of the online survey

In the first step, an online survey was used to gather global experts’ and users’ perspectives on opportunities and principal challenges presented by the characterisation of medicinal plant extracts under different laboratory infrastructure conditions. The survey responses were crucial for the development of the ConPhyMP statement, which was compiled and reviewed by the core group in several rounds. The aims and objectives were clearly explained at the beginning of the survey. In addition, those being surveyed were informed that participation was completely anonymous and voluntary. The survey population included researchers working in the fields of medicinal plants, phytochemical analysis, extract characterisation used in pharmacological, toxicological, clinical/intervention studies, and those who have a level of expertise in the field and are based in different geographical locations. The online survey comprised five main sections with 19 questions covering the following (for online survey questions, see Supplementary Table S1):

A) Participants’ demographic data, such as age, gender, the type of organisation where they work, career level, roles/positions they hold, and the countries/continent in which they are based.
B) Three questions covering type and focus of research, and type of extract used in research. The responses for this part were provided in multiple choice and free text options.
C) Five questions were allocated to determining the phytochemical techniques and/or database/software used or that respondents have access to, their preference, and the frequency of access to these techniques and software. The responses for this part were divided into multiple choice and free text options, ranking in order of preference, and one answer option.
D) Three questions covered core barriers/challenges and how to overcome existing barriers. The responses for this part were allowed in the form of multiple choice and free text options, as well as open-ended questions, where respondents could comment on the most important barrier.
E) Two final open-ended questions were included, where the respondents could comment on how to improve pharmacological, toxicological and clinical/intervention studies using medicinal plant extracts and medicinal plant research in general.

The survey was designed to take an average of 10–15 min to complete. Between July and December 2021, the survey link was distributed via a range of scientific society websites, official social media platforms (e.g., LinkedIn and Twitter), and through personal networks of academics (i.e., using the snowballing approach) as follows:

• Starting in July 2021 on the Society for the Medicinal Plant and Natural Product Research (GA) website (GA, 2021) and for the duration of the survey, including an email to all GA members.
• Botanical Safety Consortium (BSC) website (BSC, 2021) in July 2021, and circulated via email to all BSC stakeholders.
• Phytochemical Society of Europe (PSE) official Facebook group in July 2021, and emailed to PSE members.
• *Frontiers in Pharmacology* website (Heinrich and Jalil, 2021) in July 2021.
• International Natural Product Sciences Taskforce members update (INPST) in October 2021.

In addition, the survey link was shared during virtual meetings and events, namely, GA Congress^4^, British Medicine Herbal Association members meeting (BMHA), BSC stakeholders meeting, Society of Ethnopharmacology-India Congress (SFEC), USP Botanical Dietary Supplement and Herbal Medicine Expert Committee meeting (BDSHMEC).

### 3.2 Delphi Process

The initial distinction between the three main types of extracts (A, B and C) was developed by the lead author (MH), based on his experience in multiple roles over the last decades and several discussions. As a next step, a core group of experts was invited to join a panel incorporating expertise linked to phytochemical characterisation, as editors of core journals and based on their experiences in different research settings (i.e., industry and academic researchers from different global regions). From July 2021 until June 2022, this group met monthly for discussions and feedback in an iterative process, first developing the draft guidelines (covered in Tables 3 and 4) and then the manuscript (the consensus statement). The core group agreed on a final outcome of the Delphi Process. From February 2022 until May 2022 the pre-final version was made available for a wider discussion among invited experts, mostly journal editors. The process also included experts in regulatory affairs and phytochemical analysis.

### 3.3 Statistical analysis

Quantitative data were coded and analysed using the Statistical Package for Social Sciences (SPSS) version 27. Descriptive statistics were used to describe demographic data, using percentages and frequencies to express categorical variables. Qualitative data from open-ended questions were coded and analysed using the Qualitative Data Analysis (NVivo) version 2020. Thematic analysis was used to identify core themes (Braun and Clarke, 2006). Codes with common elements were grouped together under sub-themes, which were subsequently categorised under core themes. The authors examined the coherence of the data within each theme and the credibility of each theme in relation to the dataset, and ensured that there was no overlap between themes. The authors independently reviewed the themes, and a final interpretation was deduced.

## 4 Results

### 4.1 The online survey

#### 4.1.1 Participant characteristics

The online survey yielded a total of 363 responses. Of these, 35 only included demographic information and were not analysed further. Overall, the respondents were active researchers involved in projects in pharmacological, toxicological, or clinical/intervention studies (for demographic details, see Supplementary Table S2). The survey clearly achieved global coverage, although the response from Europe was particularly strong.

#### 4.1.2 Type, focus and management in medicinal plant research

Pharmacological *in vitro* experiments (n = 217, 66.2%) followed by pharmacological *in vivo* experiments (n = 134, 40.9%) and toxicological experiments (n = 101, 30.8%) were the main areas of research reported (Table 2).

**TABLE 2:** Main areas of research on medicinal plants captured in the survey (n = 328; multiple responses possible).

The main types of research (respondent could choose more than one answer) Dichotomy group tabulated at value 1	Frequency (% of cases)
Pharmacological experiments (*in vitro*)	217 (66.2%)
Pharmacological experiments (*in vivo*)	134 (40.9%)
Toxicological experiments	101 (30.8%)
Clinical/intervention studies	74 (22.6%)
Enzyme based pharmacological experiments	91 (27.7%)
Other	97 (29.6%)

Potential anti-inflammatory (n = 147, 44.8%), antioxidant (n = 140, 42.6%), and antimicrobial activity (n = 115, 35%) were the three major focus of research (see Supplementary Table S3).

Regarding the individual projects or studies managed and supervised by the respondents, overall, a relatively small number was conducted per year: “less than 5 studies” (n = 186 out of 298, 62.4%), “between 5–10 studies” (n = 89 out of 298, 29.9%), and “more than 10 studies” (n = 23 out of 298, 7.7%).

There was a general consensus that projects require phytochemical characterisation, with 53% (n = 158 out of 298) stating “always” and 24.5% (n = 73 out of 298) stating “mostly”. All other options were below 10% [“about half of the studies” - n = 18, 6%; “some of the studies” - n = 26 (8.7%), and ‘never’- n = 23 (7.7%)].

#### 4.1.3 The main extracts used in medicinal plant research

**FIGURE 1:**
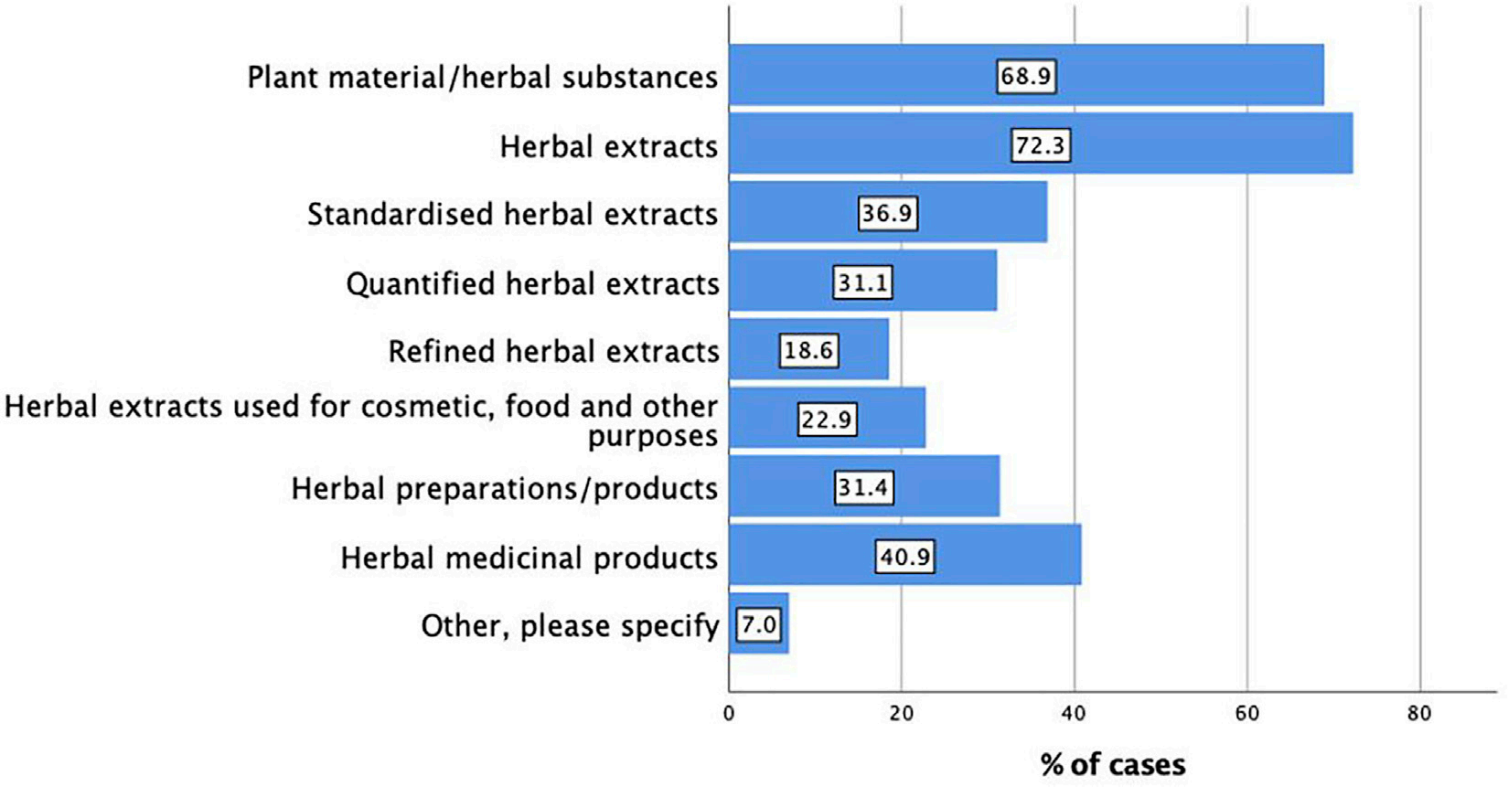
Main types of preparations used in pharmacological, toxicological and clinical/intervention studies (n = 328; multiple responses possible).

Herbal extracts (n = 273, 72.3%) followed by the plant material or herbal substances (n = 226, 68.9%) then herbal medicinal products (n = 134, 40.9%) were the main preparations used by the respondents in their research (Figure 1).

#### 4.1.4 The main phytochemical, analytical techniques or databases/software used in extract characterisation

**FIGURE 2:**
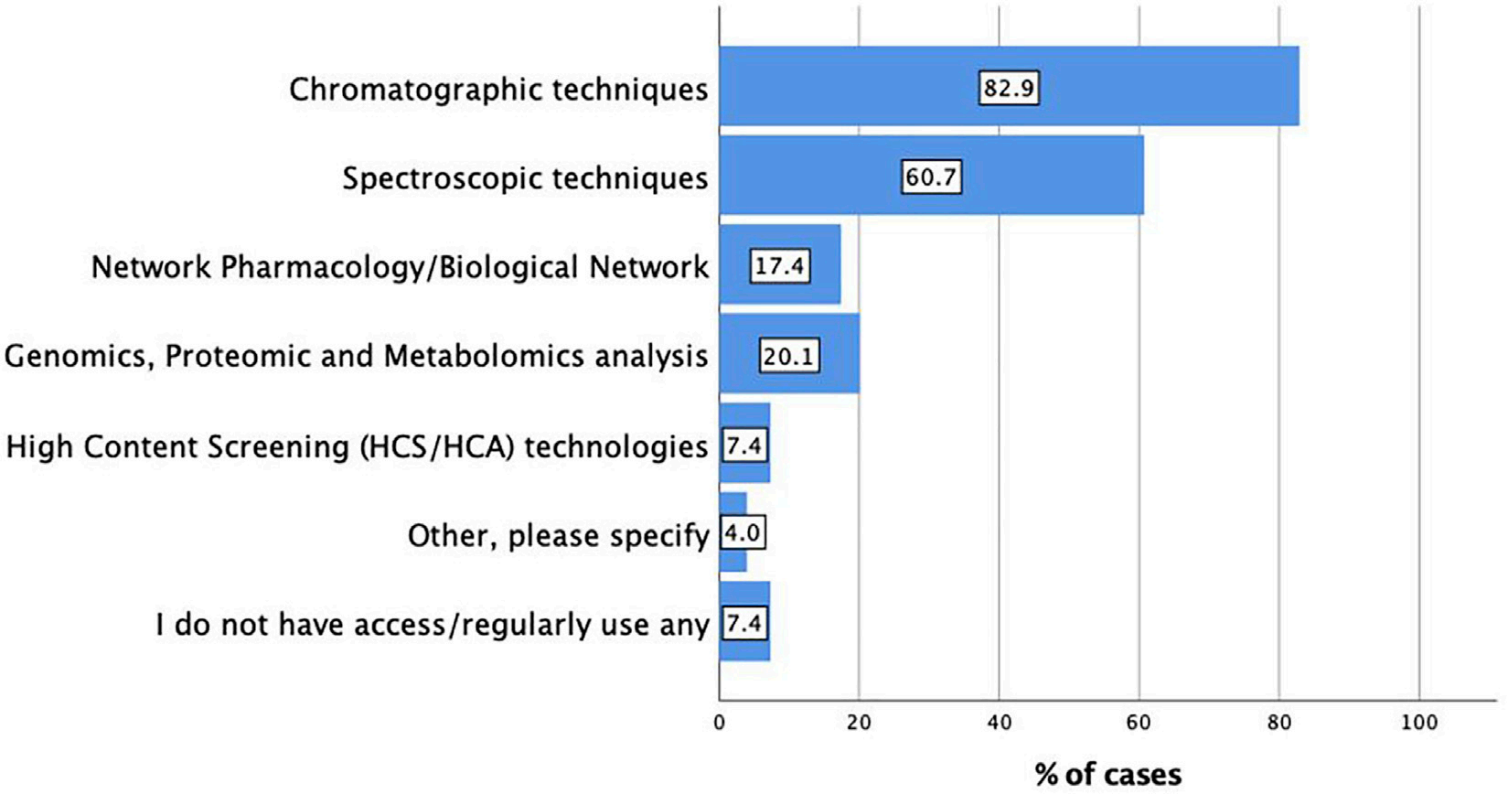
Main analytical techniques or database/software used in extract characterisation (n = 298; multiple responses possible).

The major types of analytical techniques or database/software reported were chromatographic techniques (n = 247, 82.9%), followed by spectroscopic techniques (n = 181, 60.7%). These results indicated the overarching importance of these techniques as basic tools relative to more complex techniques. Genomics, proteomics, metabolomics analysis (n = 60, 20.1%), and network pharmacology (n = 52, 17.4%) were also reported by the participants. Interestingly, only 22 respondents (7.4%) stated they do not have access to or regularly use any relevant techniques (Figure 2). As one would expect, this reinforces the call to enable access to robust and simple to use analytical techniques globally.

For frequency of access, n = 115/298 (38.6%) reported ‘always’ having access, n = 93/298 (31.2%) selected ‘most of the time’, and n = 30/298 (10.1%) said ‘about half of the time’ of having access to these techniques and software. Respondents reporting that they ‘sometimes’ or ‘never’ have access to these techniques and software numbered 41/298 (13.8%) and 19/298 (6.4%), respectively. Overall, about 80% of all respondents reported having, in principle, access to analytical tools.

Regarding preferred analytical techniques or database/software used and the respondents have access to, chromatographic techniques (n = 129, 64.5%), spectroscopic techniques (n = 107, 53.50%), and network pharmacology (n = 82, 41%) were the three major techniques and database (see Supplementary Table S4).

#### 4.1.5 Core barriers in extract characterisation

**FIGURE 3:**
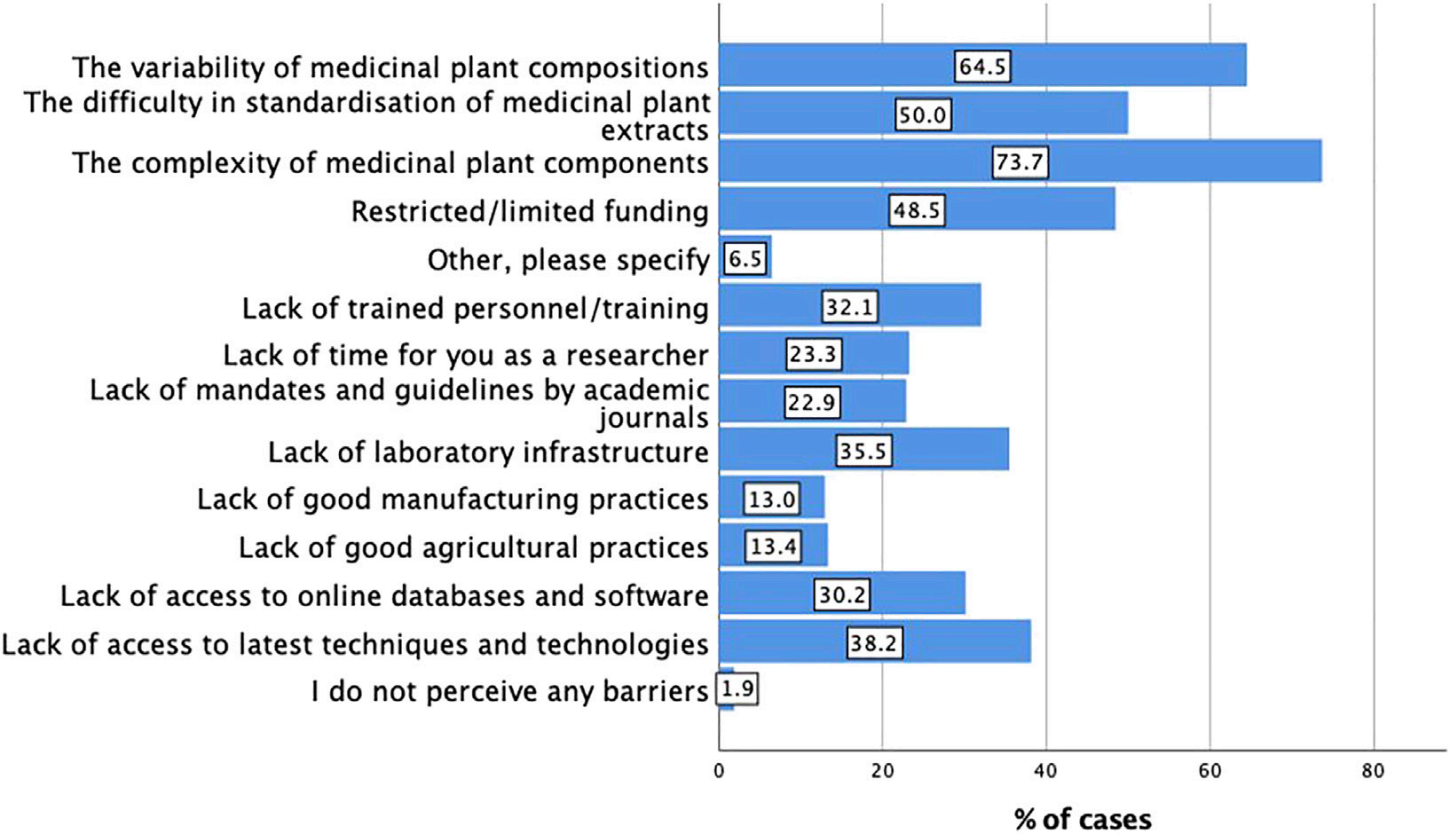
Core barriers in extract characterisation (n = 262; multiple responses possible).

The main core barriers and challenges in extract characterisation were the complexity of medicinal plant components (n = 193, 73.7%) followed by the variability of medicinal plant components (n = 169, 64.5%) followed by the difficulty in standardisation of medicinal plant extracts (n = 131, 50%). Only five respondents (1.9%) reported they do not perceive any barriers and challenges in extract characterisation. Figure 3 shows the responses to the core barriers by the respondents in the multiple choice options and free text.

#### 4.1.6 Overcoming existing core barriers in extract characterisation

**FIGURE 4:**
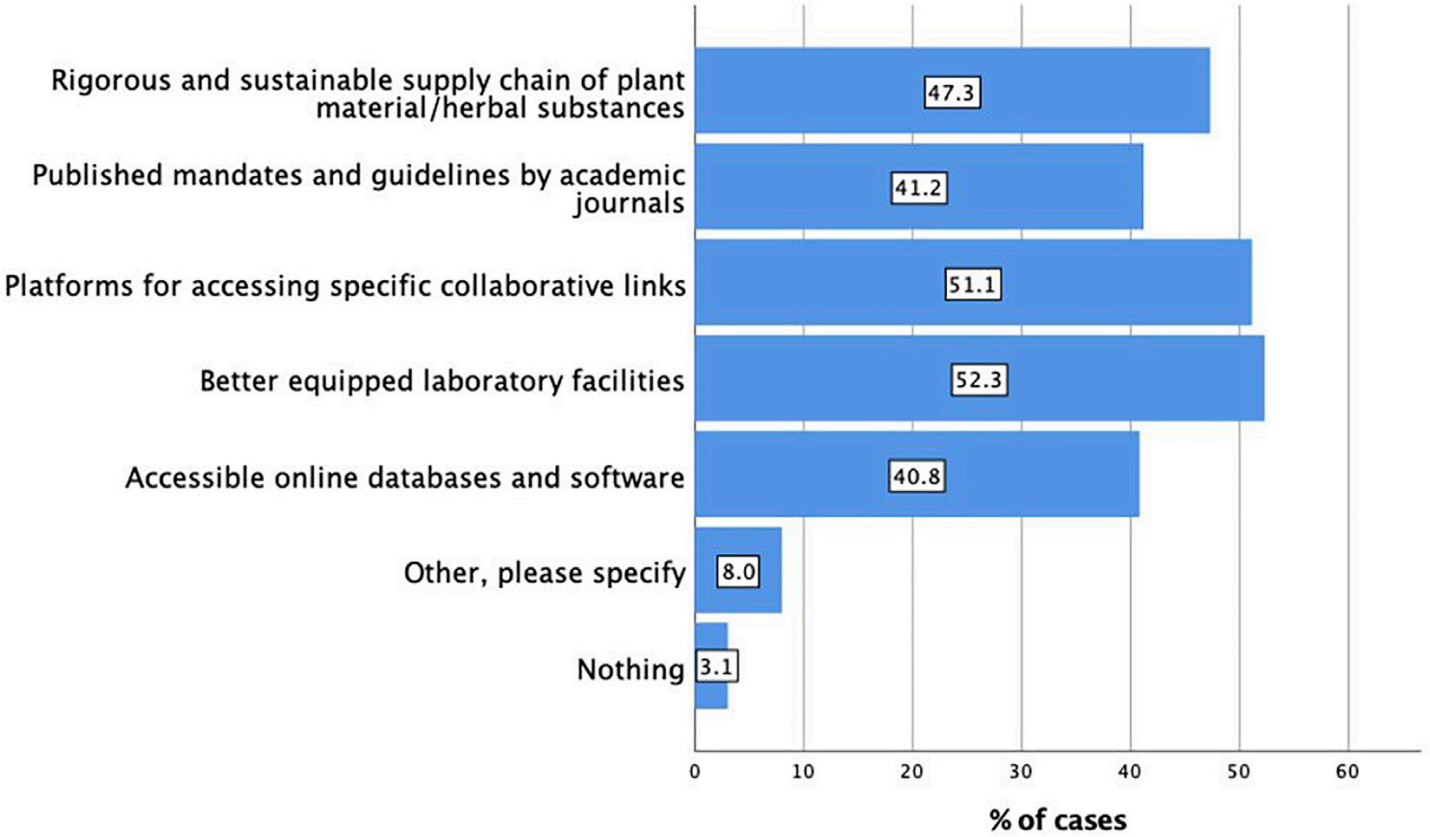
Overcoming existing core barriers in extract characterisation (n = 262; multiple responses possible).

Better equipped laboratory facilities (n = 137, 52.3%) followed by platforms for accessing specific collaborative links (n = 134, 51.1%), and then a rigorous and sustainable supply chain of plant material/herbal substances (n = 124, 47.3%), and published mandates and guidelines by academic journals (n = 108, 41.2%) were listed as the main overcoming strategies for the existing core barriers in extract characterisation. As one would expect, responses to overcoming barriers provided by the respondents in the multiple choice options and free text (Figure 4) pointed to the overarching need for better access to an analytical infrastructure/network that would enable such multidisciplinary work, and to better access of existing data.

#### 4.1.7 Medicinal plant research challenges and opportunities for improvement

**FIGURE 5:**
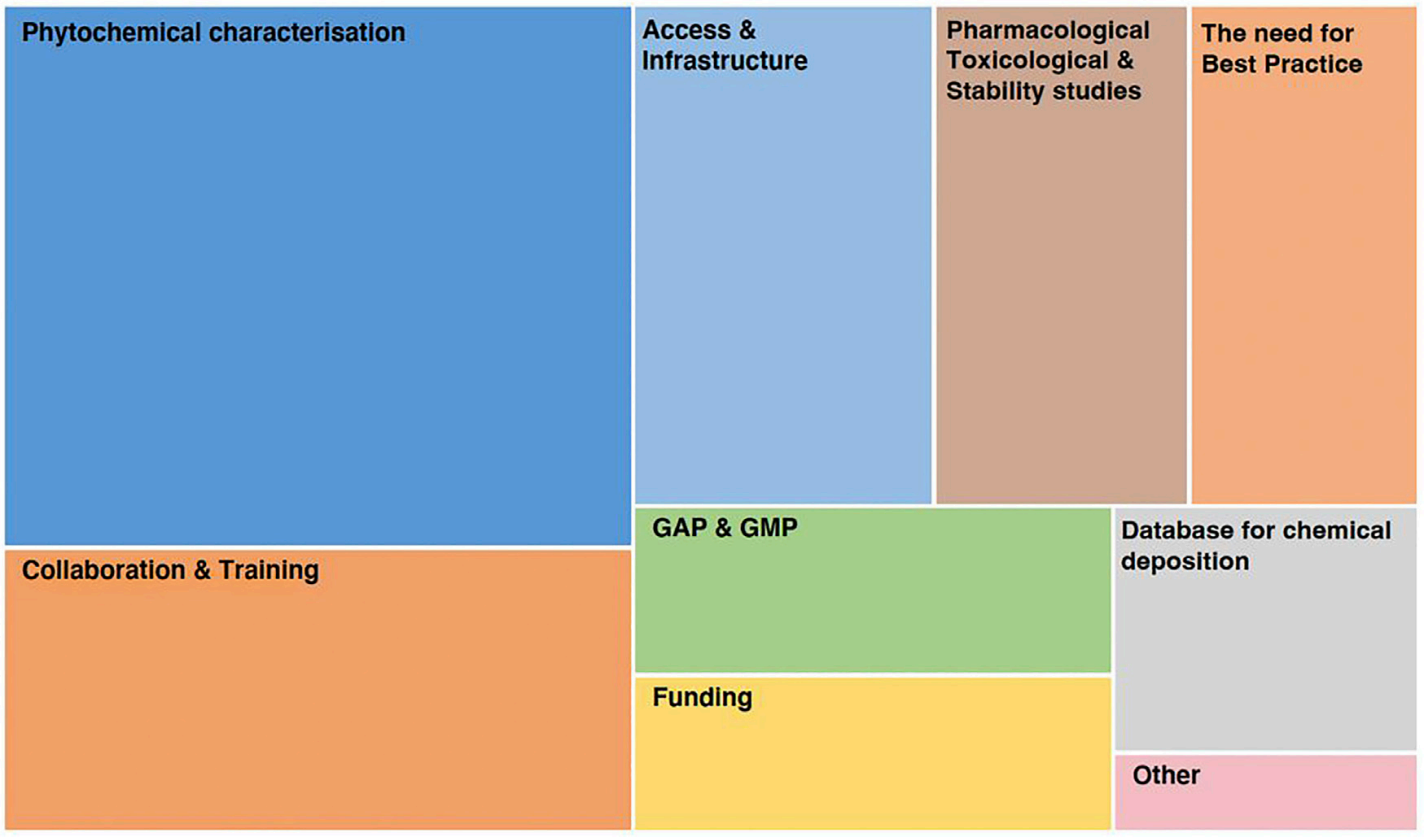
Core themes identified from open-ended questions on what can be improved in medicinal plant research.

Of those who responded to the open-ended questions about what could be improved in studies using plant extracts in particular, and in medicinal plant research in general, six core themes were identified (Figure 5). Some top areas viewed as being equally important were the need for improving the phytochemical characterisation of plant extracts, and their reporting, collaboration and training, and access to infrastructure. Improvements are needed in conducting and reporting studies using plant materials/extracts, such as pharmacological, toxicological and stability studies. This supports the need for best practice guidelines for conducting and reporting of these studies, coupled with the necessity for establishing databases for the deposition of phytochemical characterisations. Funding, Good Agricultural and Collection Practices (GACP), and Good Manufacturing Practices (GMP), were other reported areas requiring improvement (Figure 5).

#### 4.1.8 Core outcomes based on the survey

Within the research community, it is widely recognised that there is a need to improve the methods used for chemical characterisation of plant extracts in pharmacological research. Importantly, a large majority of researchers have access to infrastructure allowing such characterisation. Better equipped laboratory facilities and the need for enhanced collaboration were identified as core areas needing improvement. Clearly, there is strong motivation to support such developments.

### 4.2 The guidelines (ConPhyMP)

#### 4.2.1 Core principles and scope

The Consensus statement on the Phytochemical Characterisation of Medicinal Plant extract (ConPhyMP) statement is ideally intended to be used in all studies that investigate the activity of a plant extract, including pharmacological and toxicological, as well as clinical and interventional studies. Here, extract characterisation is essential to ensure the validity and applicability of these studies.

An important change to previous ways of approaching phytochemical analysis is the classification of extracts based on three groups (A, B and C), capturing a species importance and regulatory status. Therefore, it is not based on chemical criteria but based on the importance of a plant as a medicine (as defined by its inclusion in a pharmacopoeia) and, more generally, its importance in international trade (e.g., as a food supplement).

**FIGURE 6:**
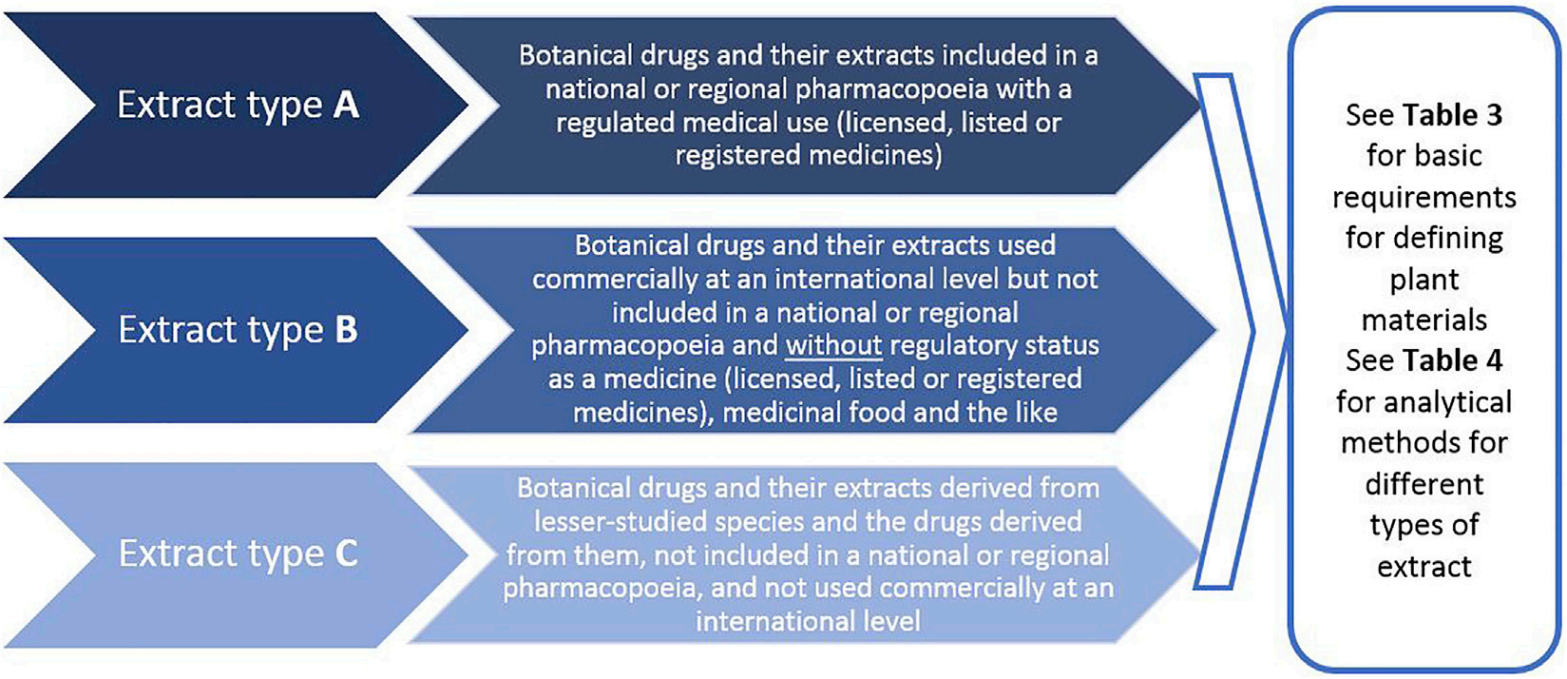
Classification of medicinal plant extract used in pharmacological, toxicological, and clinical/intervention research (see Table 3 for basic requirements for defining the plant material, and Table 4 for analytical methods of different types of extracts)—a novel way for guiding the requirements for extract characterisation.

**FIGURE 7:**
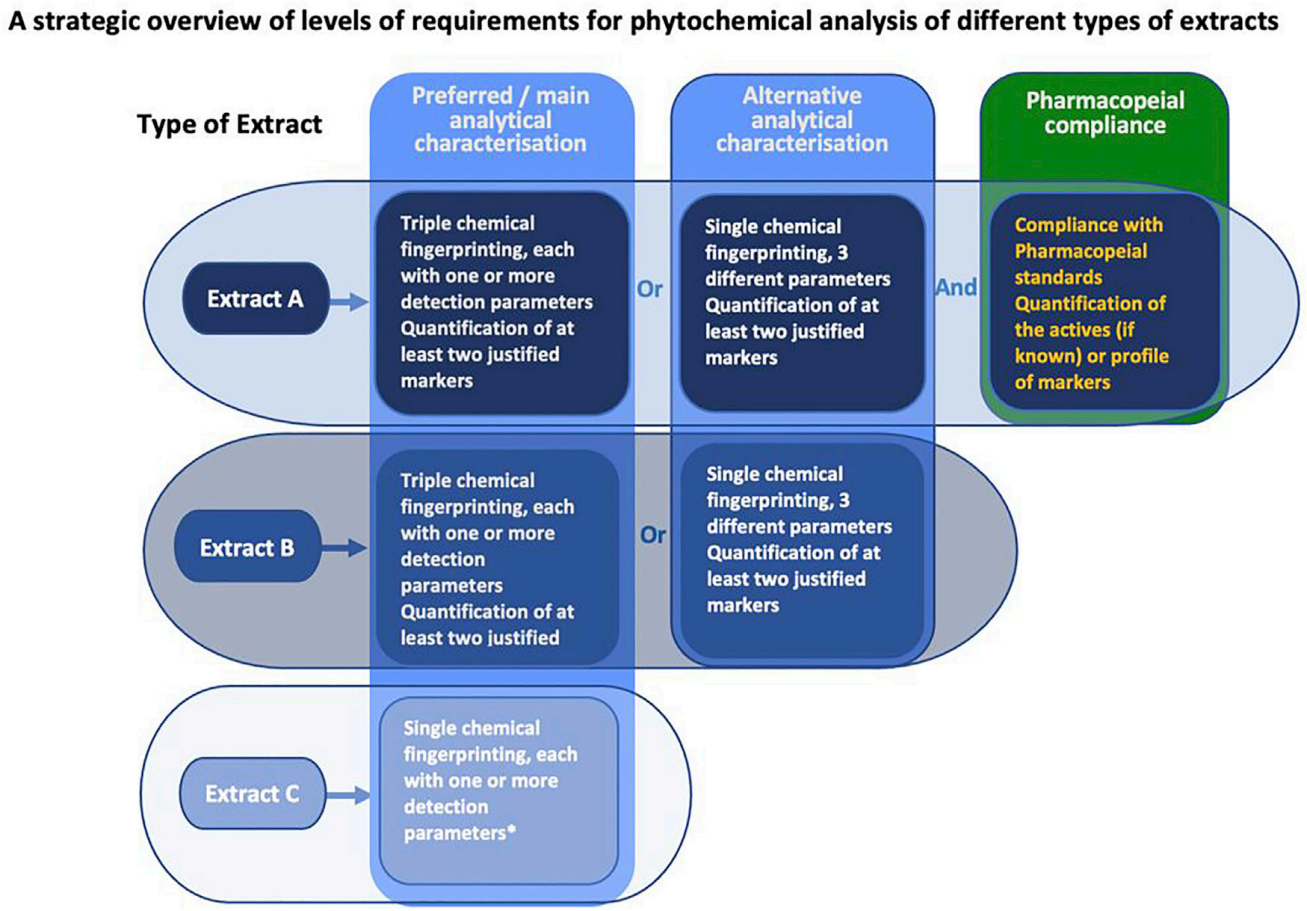
Different requirement levels for phytochemical characterisation of different extract types—a simplified overview (see Table 4 for detailed requirements of the preferred methods in extract characterisation/phytochemical analysis with their specifications) *No description of marker substances is needed but may be provided.

Specifically:

• Type A extracts include botanical drugs and their extracts *included in a national or regional pharmacopoeia* used as active ingredients in phytopharmaceuticals with a regulated medical use (licensed, listed or registered medicines);
• Type B extracts include botanical drugs and their extracts *used commercially at an international level* but not included in a national or regional pharmacopoeia and used as an ingredient for herbal preparations commercially *without regulatory status as a medicine* (licensed, listed or registered medicines), medicinal food including teas/infused drinks and the like; and
• Type C extracts include botanical drugs and their extracts derived from lesser-studied species and the drugs derived from them, which are not included in a national or regional pharmacopeia and are not used commercially at an international level (Figure 6 and Figure 7).

For each of these extract types, a different level of detail of phytochemical characterisation should be provided. However, inevitably for all types of herbal preparations, a full specification of the plant material used for extraction is desirable (see Table 3). This includes:

• Latin name and author, common/possible synonyms.
• Type of agrochemical used during the growth of the plant if possible.
• Herbal parts used (aerial parts, roots, bark, etc.), fresh, dried, fermented, etc.
• Area, season of harvesting (use of GPS coordinates for localization).
• Area, date and time of day of collection.
• Description of potential infestation (herbivory, infections with microbes and viruses, etc.).
• Compliance with the Nagoya protocol or co-authorship of institution where the plant comes from.
• Declaration of origin (natural, cultivation, plantation, etc.), and variables affecting the growth and production of bioactive compounds.
• Morphologic authentication of the plant material if possible.
• Deposition of voucher specimens.
• Information on how the plant material was authenticated and by whom.
• For combinations: exact ratios of the components.

**TABLE 3:** Recommendations (checklist of items) for reporting of the starting plant material^a^ and its initial processing and which should be used in conjunction with (Heinrich et al., 2020).

Section topic	Item number^b^	Recommendation for reporting of plant material and its initial processing
Title and abstract	1	(a) A clear and concise title including the plant material used, the experimental approach, and therapeutic focus, using a commonly understood terminology
		(b) Provide in the abstract an informative and balanced summary of what was done and what was found including implications and limitations
Description of the botanical drug and taxonomic authentication	2	Botanical or morphologic authentication of the plant material (desirable is a combination with DNA barcoding, e.g., PCR, RFLP, genome sequencing) and the information must be included in a *separate section of Material and Methods*, if applicable combined with the information required under item 3
		(a) Fully authenticated plant species name(s) based on Medicinal Plant Name Service: https://mpns.science.kew.org/mpns-portal/or the Plants of the World Online: https://powo.science.kew.org/World Flora Online: http://www.worldfloraonline.org/or another accepted taxonomic database^c^
		(b) Species name(s) as provided in a pharmacopoeia may not be taxonomically current or correct and must be checked against the above sources
		(c) Locality, date and season of harvesting and collection (including geographical coordinates, if available) should be provided
		(d) Details of voucher specimen being deposited, ideally in an institutional herbarium (including herbarium code from The William & Lynda Steere Herbarium: https://www.nybg.org/plant-research-and-conservation/explore/and accession number should be provided
		(e) Information on how the material was authenticated and by whom
		(f) Specific observations about the samples collected, e.g., particular environmental or biological conditions, if applicable
Description of the extract and extraction process	3	The following information must be included in a *separate section of Material and Methods*, if applicable combined with the information required under item 2
		(a) The full species name(s), including authorities and family, needs to be included as well as the international drug name, if one has been assigned in a pharmacopoeia. However, a drug name is no substitute for the binomial (species name)
		(b) Plant parts used (aerial parts, roots, bark, etc.), fresh, dried, fermentedetc.
		(c) Drug-solvent ratio (DSR)^d^ and resulting drug-extract ratio DER)^e^
		(d) If applicable, pre-extraction and fermentation procedures and storage conditions for the extracts and preparation used
		(e) Extraction solvent (mixture)
		(f) Type of extract by consistency (dry, liquid/fluid, soft, etc.)
		(g) Mode of extraction (percolation, maceration, etc.)
		(h) Extraction pressure, temperature, and duration
		(i) Drying mode (spray drying, freeze drying, etc.)
		If applicable
		(j) Traditional processing of the material used medicinally (fumigation, steaming, roasting, cooking, frying, etc.)
		(k) Other processing steps (liquid-liquid, provoked precipitation, etc.), purification process (resin adsorption, fractionation, etc.)
		(l) Exact ratios of the individual botanical (and other) drugs and their processing (in case of multicomponent extracts)
		(m) Details of standardisation or quantification of marker compounds
		(n) The content, including the concentration of active ingredients or marker compounds identified in the individual pharmacopoeial monographs
		(o) The name and concentration of any added antimicrobial compounds or preservatives
		(p) The list and % of any added excipients
Documentation of the legal basis for collection and processing	4	(a) Full compliance with the Nagoya protocol, CITES, and all associated treaties
		(b) Full compliance with phytosanitary regulations
		(c) Collaboration and co-authorship of the institution where the plant comes from is encouraged
In case of a finished (commercial) product: description of product characteristics	5	(a) Details about the proprietary product name, i.e., brand name
		(b) The manufacturers’ name and supplier of the product
		(c) Pharmaceutical forms (tablets, capsules, etc.), dose, frequency and duration of treatment
		(d) The amount of extract per dosage unit, if applicable
		(e) List of ingredients (including the description of extract ratios and solvents)
		(f) List and % of excipients used
		(g) Information on the products’ regulatory status, i.e., is it licensed/registered/listed in the country in which the study was conducted
		(h) Batch number and date of production/best by information
		(i) Details of storage conditions

a This article is focused on terrestrial plants and the extracts derived from them. Marine organisms and pure natural products isolated, as well as animal-derived preparations are not covered. Many of these guidelines are in principle also applicable to mushrooms (fruiting body of a fungus) especially those which are used in food or medicine or are known to be toxic, but they are not covered explicitly.

b As is common with other consensus statements, each item of the checklist is numbered for ease of use. The numbering will enable the authors, reviewers and editors to quickly evaluate and find the relevant information/core items in the submitted manuscript.

c For species name(s) of fungi see Index Fungorum: http://www.indexfungorum.org/Names/Names.asp and Species Fungorum: http://www.speciesfungorum.org/Names/Names.asp or MycoBank: https://www.mycobank.org/.

d Represents the amount of plant material used to a measured amount of extract, and is calculated by dividing the amount of herbal material by the amount of solvent used.

e Represents the amount of extract obtained from the amount of herbal drug, and it varies considerably depending on the amount of herbal drug, solvent used, extraction process, and other variables.

In addition, for herbal preparations such as extracts, information on extraction parameters has to be provided (see Table 3). This includes:

• Drug to solvent ratio, and resulting drug to extract ratio.
• Extraction solvent (mixture).
• Extraction mode (percolation, maceration, etc.).
• Extraction pressure, temperature and time.
• Type of extract by consistency (dry, liquid/fluid, soft, etc.).
• If applicable: further process steps (liquid-liquid-extraction, provoked precipitation, etc.).
• If applicable: list of any added excipients during production.

If a commercial product was used, where there is no data on one or more of the above parameters, the batch number needs to be specified and a voucher specimen needs to be deposited. In addition to the extraction parameters, the phytochemical composition needs a comprehensive assessment. Ideally, multiple analytical methods should be used *in addition* to the pharmacopoeial compliance. For herbal extracts not yet included in pharmacopoeias, these methods are essential to define a chemical fingerprint according to the state-of-the-art. However, since every detection method has its intrinsic shortcomings (Upton et al., 2020; Zhao et al., 2022), using just one method may result in detecting only a subset of constituents, leaving a large share of substances undetected. For example, thin-layer chromatography only visualizes substances that are either intrinsically fluorescent or absorbent, or, if a staining reagent was used, only substances that react accordingly. Taking the widely used HPLC-UV analysis as another example, substances are resolved within only one dimension in a specific elution window. Substances eluting outside the elution window in the “injection peak” and/or the rinse step are not resolved and cannot contribute to the fingerprint. Substances lacking a chromophore, like some terpenes, will remain undetected. The strengths and limitations of the common chemical fingerprinting methods are summarized in Table 5.

To overcome the respective shortcomings and to ensure a best practice in extract characterization according to the state-of-the-art, fingerprinting is an essential prerequisite, and, if applicable (for type A extract), in combination with pharmacopoeial compliance. We propose the use of ideally three different/orthogonal fingerprinting methods. If access to multiple methods is scarce, different/orthogonal detection parameters within one method should be applied, like different staining methods in TLC, different elution gradients, different stationary phases and/or different detection wavelengths in HPLC. Applying this workflow, a comprehensive characterization is ensured, countervailing the intrinsic limitations of the common fingerprinting methods. The fingerprinting of the active ingredients or marker compounds need to be conducted irrespective of whether they are active, toxic metabolites and/or analytical markers in the plant extract under investigation. The workflow for characterization of either regulated, non-regulated, or newly/less studied herbal extracts is summarized in Figure 7.

This strategy applies to all extracts, but clearly specific analytical approaches may be needed in some important exceptional cases. In case of essential oils, for example, due to distillation process, where only volatile compounds - mostly terpenes - are extracted, the resulting phytochemical composition of essential oils is usually less complex compared to extracts generated by extraction with a solvent. While extracts typically contain several thousands of different natural compounds, essential oils are narrowed down to some dozens. However, the same considerations basically apply here, since variation between essential oils of the same species due to, among others, seasonal variation or altered distillation parameters frequently occurs. Therefore, a comprehensive analytical characterization is of same importance like for other extracts. Due to the nature of most volatile compounds found in essential oils, some analytical methods presented here are of limited suitability for a proper analysis, e.g. HPLC-UV, due to a lack of chromophores in many monoterpenes. Also, when using TLC/HPTLC on the mainly hydrophobic constituents of essential oils, the elution solvent needs to be chosen properly in order to facilitate a thorough separation of the substances within the elution window. NMR as a method does not need any special tweaking for essential oils, whereas GC based methods are predestinated for analysis. Taking these considerations in account, a comprehensive analytical characterization is also possible and should be carried out when working with essential oils. Importantly, the guidelines apply fully, if an extract from an essential oil containing botanical drug is used using water or an organic solvent as the extractant.

#### 4.2.2 How to use ConPhyMP

The statement/checklist consists of two tables with accompanying explanatory figures. Table 3 provides recommendations for reporting of the starting plant material and its initial processing. It contains five items and is divided into the following main sections:

1) Title and abstract;

2) Description of the botanical drug and taxonomic authentication;

3) Description of the extract and the extraction process;

4) Documentation of the legal basis for collection and processing; and.

5) Description of finished product characteristics (Table 3).

Table 4 presents recommendations for conducting and reporting the most suitable analytical methods for defining the chemical profile based on the different types of extracts. It consists of five items and is divided into the following main sections:

**TABLE 4:** Recommendations (checklist of items) for most suitable analytical methods for defining the chemical profile for different types of extracts.

Section topic	Item number^a^	Recommendation for conducting and reporting of analytical methods based on types of extract		
Type of extract (see Figure 6)	1	A	B	C
Preferred/main methods for extract characterisation/chemical analysis (see Figure 7)	2	Compliance with pharmacopoeial standards to be followed: (a) Description of the active ingredients in the botanical drug (if known) or analytical marker compounds as defined	Not applicable for extract type B	Not applicable for extract type C
		(b) An analysis as defined in the monograph is needed if the extract has not been supplied with a certificate	Not applicable for extract type B	Not applicable for extract type C
		(c) If the preparation was purchased, manufacturer and certificate of analysis need to be included	Not applicable for extract type B	Not applicable for extract type C
		Including either the preferred or alternative approaches for characterisation: (a) Triple chemical fingerprinting methods, each with one or more detection parameters	(a) Triple chemical fingerprinting methods, each with one or more detection parameters	Not applicable for extract type C
		(b) Quantification of at least two marker compounds (unless this is not feasible evidence needs to be provided), and justification of the choice of markers (if applicable)	(b) Quantification of at least two marker compounds (unless this is not feasible evidence needs to be provided), and justification of the choice of markers (if applicable)	Not applicable for extract type C
Alternative methods for extract characterisation/chemical analysis (see Figure 7)	3	(a) Single chemical fingerprinting methods with at least three different detection parameters (i.e., altered detection parameters, like TLC/HPTLC with different derivatization conditions, HPLC-DAD/LC-DAD with different wavelengths). The same applies to coupling MS or NMR to chromatographic techniques	(a) Single chemical fingerprinting method, three different detection parameters should be provided (as in A)	(a) Single chemical fingerprinting methods with three different detection parameters (as in A)
		(b) Quantification of at least two marker compounds (unless this is not feasible evidence needs to be provided), and justification of the choice of markers (if applicable)	(b) Quantification of at least two marker compounds (unless this is not feasible evidence needs to be provided), and justification of the choice of markers (if applicable)	No description of marker substances is needed but may be provided
Use of reference standards	4	(a) Direct overlay of the chromatogram of the sample with that of the official individual reference standards of the marker compounds	As in extract type A	As in extract type A
		(b) Chromatographic fingerprinting: Direct overlay of the chromatogram of the sample with that of official reference standards of the powdered plant material or the dry extract from the plant material	As in extract type A	As in extract type A
Comparison of different extract/samples of the same plants	5	(a) Direct comparison of the chromatographic/spectroscopic system and/or scoring system for “similarity” to be followed	As in extract type A	As in extract type A

a As is common with other consensus statements, each item of the checklist is numbered for ease of use. The numbering will enable the authors, reviewers, and editors to quickly evaluate and find the relevant information/core items in the submitted manuscript.

1) Types of extracts, which are subdivided into three defined types (see Section 4.2.1) (Figure 6 and Figure 7).

2) Preferred/main methods for extract characterisation based on the type of extract used in the studies. Specifically, the requirement for conducting and reporting of the analytical methods for extract characterisation of extract A is more stringent than for extract B, and characterization of extract B is more stringent than for extract C;

3) Alternative methods for extract characterisation based on the type of extract used (Figure 6 and Figure 7);

4) Use of reference standards; and.

5) Comparison of different extracts/samples of the same plants.

In each of the tables, there is a list of ‘items’, which help the user to assess whether she or he complies with the requirements. In this regard, the tables can be used as a checklist, confirming that a manuscript has been checked based on the ConPhyMP statement. Users of the checklist also need to consult the detailed requirements on the scientific nomenclature of plants (Rivera et al., 2014), ethnopharmacological field studies (Chan et al., 2012; Heinrich et al., 2018), and phytopharmacological studies (Heinrich et al., 2020) to ensure the appropriate interpretation of each item description, and its relevance to the studies being conducted.

The analytical techniques applied worldwide for extract characterization differ greatly depending on the laboratory equipment available. Hence, in less-well provided laboratories, mostly TLC/HPTLC methods are used which deliver data with lower resolution compared to HPLC and GC. In fact, HPLC-UV and GC-FID may be generally considered suitable for detailed characterization of extracts as long as they are adequately validated. Apart from their high availability throughout the world, they deliver also easy to interpret data. Of course HPLC-MS and GC-MS represent highlight techniques that are characterized by very high sensitivity and resolution and should be preferably applied whenever possible. But in case they are not available, HPLC-UV and GC-FID are quite sufficient for generating informative fingerprints. NMR spectroscopy may provide high structural information but it requires specially trained personnel, is very expensive and suffers from low sensitivity. Therefore, it is not considered the method of choice for extract characterization. Also ATR-IR is rather limited in its resolution and is not suitable for complex mixtures. For a better overview on the advantages and limitations of the individual analytical techniques see Table 5.

**TABLE 5:** Strengths and limitations of the most suitable analytical methods for defining the chemical profile for different types of extracts based on the current state-of-the-art.

Fingerprint method	Strengths	Limitations	Why multiple detection is necessary^a^
TLC/HPTLC	- inexpensive	- only for intrinsic fluorescent/(UV-) absorbing or specifically stainable substances	Due to specific staining, only subsets of substances are visible within one chromatogram
	- high availability throughout the world	- therefore: intensities may be misleading	- fluorescent/(UV-) absorbing compounds with UV light
	- easy to conduct and to interpret data	- only substances within the elution window are resolved	- alkaloids/amines with Dragendorff’s reagent
	- all-round method	- lower resolution compared to HPLC and GC.	- unsaturated compounds with iodine vapour
	- relatively fast due to possibility of parallel analysis		- nucleophilic compounds with *p*-anisaldehyde/H_2_SO_4_
			- phenolic compounds with FeCl_3_, Marquis, Gibbs reagent
HPLC-UV	- medium resolution	- co-elution possible	Different detection wavelengths are necessary to detect different classes of compounds
	- medium sensitivity	- only substances within the elution window are resolved	- double bonds at 205–220 nm
	- all-round method	- only substances with chromophores are detected	- phenolic moieties commonly at 254 nm
	- quantification possible	- low structural information	- flavones, flavonols at 340–360 nm
	- high availability throughout the world		- higher conjugated aromatic or polyenic moieties at > 400 mn
	- relatively easy to interpret data		
HPLC-MS	- very high sensitivity	- not all compounds ionize	Since some compounds ionize only in positive **or** negative ion mode, both ion modes should be applied to detect as much as possible. Some substances may need different ionization sources, like ESI, APCIetc.
	- very high resolution (resolution of co-eluting substances based on mass identification)	- expensive method	
	- quantification possible in targeted approach, can be misleading in untargeted fingerprinting	- specially trained personnel necessary for interpretation of data	
	- medium structural information		
GC-FID	- high resolution	- only suitable for compounds, which are volatile, or which are able to be derivatized	To exclude co-elution of interfering substances, different temperature gradients and stationary phases may be applied
	- absolute quantification of all compounds	- low structural information	
		- co-elution of interfering compounds possible	
		- only substances within the elution window are resolved	
GC-MS	- very high resolution	- only suitable for compounds, which are volatile, or which are able to be derivatized	Since some compounds ionize only in positive **or** negative ion mode, both ion modes should be applied to detect as much as possible. Some substances may need different ionization sources, like EICI.
	- quantification possible in targeted approach, can be misleading in untargeted fingerprinting	- only substances within the elution window are resolved	
	- high structural information (NIST database)		
NMR	- all organic compounds are detected	- low sensitivity	To obtain more detailed structural information,^13^C in combination with ^1^H NMR spectra should be generated. ^1^H NMR spectra are characterized by relatively high sensitivity but low resolution. In contrast,^13^C NMR spectra provide lower sensitivity and high resolution
	- high structural information	- expensive, maintenance intensive method	
	- medium resolution	- specially trained personnel necessary for the interpretation of the data	
	- quantification possible with qNMR.	- low availability throughout the world	
		- difficult to apply in complex mixtures	
ATR-IR	- all organic compounds are detected	- low resolution	
	- medium structural information	- low availability throughout the world	
	- medium sensitivity	- not widely recognized fingerprinting method	
	- very fast method	- not applicable for mixtures	

a Specified properties of one method are considered relative to all the other listed methods. HPLC is medium resolution relative, e.g., to LC-MS or GC-MS.

## 5 Discussion

The overarching objective of this communication is to provide criteria that need to be considered during the conduct and subsequent review of investigations performed with various forms of terrestrial plant materials, especially those touted to be of value to humans. Of course, the myriad of natural remedies falls into various categories in terms of the stage of development, and the extent of characterization should vary based on the position occupied by the preparation from a species/botanical drug on this spectrum. Taking this into account, we present our view of ‘best practices’, based on the current state-of-the-art, which certainly will need revision following future advancements in science in general, and analytical methods in particular. The consensus is based upon a survey of over 300 investigators and users, although it remains unclear how representative the results of the survey are. It is recognised that not all investigators will be in a position to conform to what we present as ‘best practice’, and others may simply consider this point-of-view as moot. Additionally, manufacturers of proprietary products may not want to disclose their intellectual property. However, the very positive feedback from editors and other key stakeholders highlights the need for such guidelines and we are certain that more journal editors and other groups of experts will support these recommendations.

So, what is the value of this process and global discussion? In some cases, where investigators do not have the ‘where-with-all’ to follow what we describe as ‘best practice’, we hope the long-term achievement of implementing such an approach will be viewed as aspirational. In every case, however, it should be incumbent on the investigator to convincingly describe how others can reliably reproduce the reported results, and how others can reliably formulate and test new hypotheses based on the work. Otherwise, the report risks being classified simply as an anecdote.

## 6 Conclusion

The present guidelines (the consensus statement) are a ‘first of its kind’. We recognise the importance of best practice in publishing guidelines and these offer scientists a clear framework based on the state-of-the-art. This is well exemplified by the various versions of the Consort statement (Consolidated Standards of Reporting Trials), which since 1996 has provided a standard means for authors to prepare reports of trial findings (Begg et al., 1996). Here, we do not suggest a standard way of reporting, but define what needs to be reported in order to ascertain reproducibility. Using the ConPhyMP guidelines will hopefully improve the reproducibility of phytopharmacological research, and, at the same time, they will need to be continuously scrutinized and developed. The division of plant extracts into three different levels should help to overcome the essential concerns of many in the scientific community that research teams cannot comply with only one set of perfect and uniform standards. As such, the statement is also a call for more interdisciplinary collaboration and ultimately to develop transdisciplinary approaches. Based on the results of the survey, further training in how to integrate such tools into pharmacological research is vital.

There remain some important limitations. The feedback has been global and we have attempted to integrate the views of all who responded, but there cannot be a complete consensus between all. The intrinsic complexity of plant extracts makes it difficult to define universal principles. However, we trust that the basic approach, as defined here, will help to sharpen the scientific discussion on how to achieve improvements in other fields like marine natural product research or natural product drug discovery. In contrast to academic researchers, manufacturers of proprietary products may still be hesitant in disclosing their intellectual property.

Since our approach aims to countervail the intrinsic shortcomings of analytical methods, superordinate shortcomings of our approach need to be acknowledged. There are some important compounds that may be present in plant extracts which are still not sufficiently covered by our presented approach (see also Section 4.2.1 on essential oils). These include natural polymers, inorganic constituents and pesticides in particular, and substances in trace amounts in general. In addition, potential adulteration often needs more tailored methods than can be provided by untargeted fingerprinting. Some argue that future developments will require deeper and deeper phytochemical profiling of the extracts. This is especially critical information when evaluating the safety of the extract. Consequently, for some types of commercial products, the proposed requirements may well be considered too preliminary. Covering these classes of substances as well is beyond the scope of our presented standards, and beyond the scope of possibilities for many researchers.

We look forward to further development of these concepts. ConPhyMP will only thrive if it continuously evolves based on global use and robust debate, leading to refinement of the proposed methods and classifications. We plan to facilitate this by making a decision tool available to assist in defining what type of analysis is best suited for a given preparation to be studied (for details see https://ga-online.org/bestpractice/). This site will serve as a platform for updates and further developments. Accordingly, we look forward not only to implementation, but also to changes and further developments of the concepts presented herein.

## Data Availability

The original contributions presented in the study are included in the article/Supplementary Material, further inquiries can be directed to the corresponding author.

## Footnotes

3 Of note, simple spot tests in combination with staining are not acceptable for characterising extracts, since they are not a reliable tool to identify classes of compounds and are far too non-specific.

4 69th International Congress and Annual Meeting (2021) of the Society for Medicinal Plant and Natural Product Research (GA) held virtually and organised by Prof. Werner Knoess and collaborators at the University of Bonn, Germany.

## Abbreviation

ATR-IR, attenuated total reflection-infrared spectroscopy; GC-FID, gas chromatography-flame ionization detection; GC-MS, gas chromatography-mass spectrometry; HPLC-MS, high performance liquid chromatography-mass spectrometry; HPLC-UV, high performance liquid chromatography-ultraviolet; HPTLC, high performance thin layer chromatography; LC-MS, liquid chromatography-mass spectrometry; NMR, nuclear magnetic resonance; TLC, thin layer chromatography; SPSS, statistical package for social sciences.

## Appendix: The ConPhyMP advisory group

The following individuals are members of the ConPhyMP Advisory group who provided input on the ConPhyMP guidelines and the MS and consisted of:

- **Appendino, Giovanni**, *Fitoterapia*, Editor in Chief/Dipartimento di Scienze del Farmaco, Largo Donegani 2, 28100 Novara, Italy;
- **Arroo, Randolph**, Phytochemical Society of Europe, Committee member/Leicester School of Pharmacy, Hawthorn Building (HB2.31), the Gateway, Leicester LE1 9BH, De Montfort University, United Kingdom;
- **Atanasov, Atanas G**, *Current Research in Biotechnology,* Editor in Chief/International Natural Product Science Taskforce (INPST)/Ludwig Boltzmann Institute for Digital Health and Patient Safety, Medical University of Vienna, Spitalgasse 23, 1,090 Vienna, Austria, Institute of Genetics and Animal Biotechnology of the Polish Academy of Sciences, Jastrzebiec, 05–552 Magdalenka, Poland;
- **Barron, Denis**, Groupe Polyphénols, President/Nestlé Institute of Food Safety and Analytical Sciences, Société des Produits Nestlé S.A., Lausanne, Switzerland;
- **Bauer, Rudolf,** Good Practice in Traditional Chinese Medicine Research Association (GP-TCM), founding president and current board of director/Institute of Pharmaceutical Sciences, Department of Pharmacognosy, University of Graz, Graz, Austria;
- **Cañigueral, Salvador**, European Pharmacopoeia Commission, Chair/Unitat de Farmacologia, Farmacognòsia i Terapèutica, Facultat de Farmàcia i Ciències de l’Alimentació, Universitat de Barcelona, Av. Joan XXIII, 27–31, ES-08028, Barcelona, Spain;
- **Efferth, Thomas**, *Phytomedicine*, Editor in Chief/Institute of Pharmaceutical and Biomedical Sciences, Chair, Department of Pharmaceutical Biology, Johannes Gutenberg University Staudinger Weg 5, 55128 Mainz, Germany;
- **Fürst, Robert**, *Planta Medica*, Editor in Chief/Institute of Pharmaceutical Biology, Goethe University Frankfurt, Max-von-Laue-Str. 9, 60438 Frankfurt, Germany;
- **Izzo, Angelo A**, *Phytotheraphy Research*, Editor in Chief/Department of Pharmacy, School of Medicine and Surgery, University of Naples Federico II, Via D Montesano 49, Naples, Italy;
- **Kelber, Olaf** Society of Medicinal Plants and Natural Product Research (GA) secretory/Bayer Consumer Health, Steigerwald Arzneimittelwerk GmbH, Darmstadt, Germany;
- **Kemper, Kathi**, *Complementary Therapies in Medicine,* Editor in Chief/Ohio State University College of Medicine, 43210–1,238, Columbus, Ohio, United States;
- **Monagas, Maria,** Principal Scientist/Dietary Supplements and Herbal Medicines, United States Pharmacopoeia, 12601 Twinbrook Parkway, Rockville, MD, 20852, United States;
- **Pendry, Barbara**, *Journal of Herbal Medicine*, Editor in Chief/Medicines Research Group, School of Health, sport and Bioscience, University of East London, London E15, United Kingdom;
- **Pereda-Miranda, Rogelio**, *Brazilian Journal of Pharmacognosy* (Springer), Editor in Chief/Universidad Nacional Autónoma de México, Ciudad de México, Mexico;
- **Rollinger, Judith**, Society of Medicinal Plants and Natural Product Research (GA), President/Division of Pharmacognosy, Department of Pharmaceutical Sciences, University of Vienna, Althanstr. 14, room 2 F 449, 1,090 Vienna, Austria;
- **Russo, Alessandra**, Italian Society of Pharmacognosy (SIPHAR), President/Department of Drug and Health Sciences, University of Catania, V. le A. Doria 6, 95125 Catania, Italy;
- **Verpoorte, Robert**, *Phytochemistry Reviews*, Editor in Chief/Natural Products Laboratory, Institute of Biology, Leiden University, P.O BOX 9505, 2300RA Leiden, The Netherland;
- **Visioili, Francesco**, *PharmaNutrition*, Editor in Chief, and *Pharmadvances*, ​Executive Editor/Department of Molecular Medicine, University of Padova, Padova, Italy, IMDEA-Food, CEI UAM + CSIC, Madrid, Spain;
- **Vollmer, Günter**, International Society for Ethnopharmacology (ISE), President/Institute of Zoology, Molecular Cell Physiology and Endocrinology, University of Dresden, Dresden, Germany;
- **Wohlmuth, Hans**, formerly Head of Research and Development at Integria Healthcare, Eight Mile Plains, Queensland, Australia/NICM Health Research Institute, Western Sydney University and School of Chemistry and Molecular Biosciences, University of Queensland, Australia.
