# Supplementary material for: Best Practice in the chemical characterisation of extracts used in pharmacological and toxicological research—The ConPhyMP—Guidelines
Source: Front Pharmacol. 2022 Sep 13;13:953205. doi: 10.3389/fphar.2022.953205 (PMC9514875; doi:10.3389/fphar.2022.953205)
Supplement: Supplementary file 1 [file Table4.pdf]

TABLE S4 Preferred analytical techniques or software/database the respondents have access to/regularly use in their research (n=200).

**Preferred type of phytochemical and/or database used**	**1st choice**	**2nd choice**	**3rd choice**	**4th choice**	**5th choice**	**Other**
Chromatographic techniques	129 (64.5%)	53(26.5%)	8 (4.0%)	5 (2.5%)	4 (2.0%)	1 (0.5%)
Spectroscopic techniques	33 (16.5%)	107 (53.5%)	34 (17.0%)	15 (7.5%)	10 (5.0%)	1 (0.5%)
Network Pharmacology/ Biological Network	16 (8.0%)	16 (8.0%)	82 (41.0%)	47 (23.5%)	35 (17.5%)	4 (2.0%)
Genomics, Proteomic and Metabolomics analysis	16 (8.0%)	14 (7.0%)	45 (22.5%)	88 (44.0%)	30 (15.0%)	7 (3.5%)
High Content Screening (HCS/HCA) technologies	5 (2.5%)	9 (4.5%)	25 (12.5%)	44 (22.0%)	114(57.0%)	3 (1.5%)
Other	1 (0.5%)	1 (0.5%)	6 (3.0%)	1 (0.5%)	7 (3.50%)	184 (92.0%)

Measured on 5-point rank order: 5=Least preferred; 1=Most preferred.
