# Supplementary material for: Best Practice in the chemical characterisation of extracts used in pharmacological and toxicological research—The ConPhyMP—Guidelines
Source: Front Pharmacol. 2022 Sep 13;13:953205. doi: 10.3389/fphar.2022.953205 (PMC9514875; doi:10.3389/fphar.2022.953205)
Supplement: Supplementary file 2 [file Table1.pdf]

TABLE S1: Best Practice in Medicinal Plant Extract Characterisation online survey questions.

Participant Information Sheet (PIS) Title of Study: **Best Practice in Medicinal plant Extract Characterisation** Department: **UCL School of Pharmacy** Name of the **Principal Investigator: Prof. Michael Heinrich**. Name and Contact Details of the **Investigator: Dr. Banaz Jalil** (b.jalil@ucl.ac.uk). 1. Invitation Paragraph We would like to invite you to participate in this survey. Before you decide whether you want to take part, it is important for you to read the following information. Please ask us if there is anything that is not clear or you would like more information. Take time to decide whether or not you wish to take part. 2. What is the study's purpose? Our study focuses on defining Best Practice Guidelines for conduction and reporting extract characterisation used in pharmacological, toxicological and clinical/intervention studies. It is for researchers actively involved in projects in such studies. Importantly, we also plan to assess feasibility under different laboratory conditions for this type of phytochemical analysis. 3. What are the possible benefits of taking part? Your views in your role as researchers are essential. We want to gather perspectives, opportunities, and overarching challenges faced in the characterisation of medicinal plant extracts under different laboratory infrastructures globally. Your opinions will help to develop Best Practice Guidelines on how to design, conduct and report the phytochemical analysis in a reproducible and transparent way, as well as defining the requirements needed for defining the plant material/herbal substances, herbal extracts, and herbal medicinal products used in pharmacological, toxicological, and clinical/intervention studies. 4. Will my taking part in this project be kept confidential? No personal identifiable data will be collected. All of the data we collect will be confidential – there will be a participant number attached to your results. However, we will not be able to link this back to your name. **5. Contact for further information** If there is anything that is not clear, or if you would like more information, please do not hesitate to contact us: **Prof. Michael Heinrich and Dr. Banaz Jalil** (please see above). **6. The project** is conducted by a collaborative team of researchers including senior editors of leading journals in the field and is funded through a charitable donation (Fa. Schwabe). Thank you for reading the participant information sheet and for considering taking part in this study**.**

○ Yes, I have read the participant information sheet, and I agree to participate in the survey study

Page Break

End of Block: Participant Information Sheet (PIS)

Start of Block: Demographic information

Q1 What is your age group?

▼ 21-30 ... Prefer not to say

Q2 How do you describe your gender?

▼ Male ... Prefer not to say

Q3 What is the type of organisation do you work in?

- ○ Academia/University
- ○ Government
- ○ Industry
- ○ Independent Research Centre
- ○ Other, please specify ____
- ○ Prefer not to say

Q4 What is your career level?

- ◯ Ph.D. student/researcher
- ◯ Postdoctoral researcher
- ◯ Lecturer
- ◯ Senior Lecturer/Reader
- ◯ Professor
- ◯ Other, please specify ____
- ◯ Prefer not to say

Q5 Which of the following best describes your current role/position? Please select all that apply.

- ▢ Director/Head of Institute/Department
- ▢ Editor In Chief/Associate Editor
- ▢ Director/Head of Research Centre
- ▢ Head of Research group
- ▢ Manager/Supervisor
- ▢ Consultant
- ▢ Researcher
- ▢ Other, please specify ____
- ▢ I do not hold any position
- ▢ Prefer not to say

Q6 Where are you located? e.g., the Continent/Country you are based in*
(*) The division of the countries and territories of the world into regional and subregional groups used is based on the United Nations geoscheme system. It was devised by the United Nations Statistics Division (UNSD) based on the M49 coding classification (please see above link).

- ○ Africa - Northern Africa
- ○ Africa - Sub-Saharan Africa
- ○ Americas - Latin America and the Caribbean
- ○ Americas - Northern America
- ○ Asia - Central Asia
- ○ Asia - Eastern Asia
- ○ Asia - South-eastern Asia
- ○ Asia - Southern Asia
- ○ Asia - Western Asia
- ○ Europe - Eastern Europe (including Northern Asia
- ○ Europe - Western Europe (including the Mediterranean and Northern Europe)
- ○ Oceania - Australia and New Zealand

End of Block: Demographic information

Start of Block: Type, and Focus of research as well as Type of extracts used

Q7 What type of research concerning medicinal plants do you conduct? Please select all that apply.

- ☐ Pharmacological experiments (in vitro)
- ☐ Pharmacological experiments (in vivo)
- ☐ Enzyme based pharmacological experiments
- ☐ Toxicological experiments
- ☐ Clinical/intervention studies
- ☐ Other, please specify ____

Q8 What is the main focus of your research in terms of pharmacological, toxicological and/or clinical/intervention studies? Please select all that apply.

- ☐ The gastrointestinal and biliary system
- ☐ The cardiovascular system
- ☐ The respiratory system
- ☐ The central nervous system
- ☐ The endocrine system
- ☐ The reproductive tract
- ☐ The musculoskeletal system
- ☐ The skin
- ☐ The eye
- ☐ Ear, nose and oropharynx
- ☐ Weight loss
- ☐ Aphrodisiac
- ☐ Stress management
- ☐ Cosmetics
- ☐ Psychoactive
- ☐ Hallucinogen
- ☐ Infectious diseases

- ☐ Anti-cancer activity
- ☐ Antimicrobial activity
- ☐ Cytotoxicity activity
- ☐ Antioxidant activity
- ☐ Anti-inflammatory activity
- ☐ Anti-ageing
- ☐ Other, please specify ____

Q9 What is the main type of extract that you regularly use in your research? Please select all that apply.

- ☐ Plant material/herbal substances
- ☐ Herbal extracts
- ☐ Standardised herbal extracts
- ☐ Quantified herbal extracts
- ☐ Refined herbal extracts
- ☐ Herbal extracts used for cosmetic, food and other purposes
- ☐ Herbal preparations/products
- ☐ Herbal medicinal products
- ☐ Other, please specify ____

End of Block: Type, and Focus of research as well as Type of extracts used

---

Start of Block: Type, Accessibility, and Use of phytochemical techniques, and/or databases

Q10 How many individual projects/studies concerning medicinal plants do you regularly manage/supervise/conduct per year?

- ○ Less than 5 studies
- ○ 5 - 10 studies
- ○ More than 10 studies

Q11 For how many of these projects/studies do you conduct a phytochemical characterisation of the extracts?

- ◯ Always (all of the studies)
- ◯ Most of the studies (for about three-quarters of extracts studied)
- ◯ About half of the studies
- ◯ Some of the studies (for about a quarter of extracts studied)
- ◯ Never (none of the studies)

Q12 What is the main type of phytochemical/analytical techniques and/or databases/software you have the access to/regularly use? Please select all that apply.

- ▢ Chromatographic techniques
- ▢ Spectroscopic techniques
- ▢ Network Pharmacology/Biological Network
- ▢ Genomics, Proteomic and Metabolomics analysis
- ▢ High Content Screening (HCS/HCA) technologies
- ▢ Other, please specify ____
- ▢ I do not have access/regularly use any

Q13 How often do you access to the above phytochemical/analytical techniques and/or databases/software?

- ○ Always
- ○ Most of the time
- ○ About half the time
- ○ Sometimes
- ○ Never

Q14 What is your preferred type of phytochemical/analytical technique and/or database/software you have access/regularly use in your research? Please rank them in order of accessibility/usage from 1 (most preferred) to 5 (least preferred)
______ Chromatographic techniques
______ Spectroscopic techniques
______ Network Pharmacology/Biological Network
______ Genomics, Proteomic and Metabolomics analysis
______ High Content Screening (HCS/HCA) technologies
______ Other, please specify

End of Block: Type, Accessibility, and Use of phytochemical techniques, and/or databases

Start of Block: Challenges and Barriers for medicinal plant extract characterisation

Q15 What do you see (if any) as core barriers to extract characterisation in pharmacological, toxicological and/or clinical/intervention studies? Please select all that apply.

- ▢ The complexity of medicinal plant components
- ▢ The variability of medicinal plant compositions
- ▢ The difficulty in standardisation of medicinal plant extracts
- ▢ Restricted/limited funding
- ▢ Lack of mandates and guidelines by academic journals
- ▢ Lack of good agricultural practices
- ▢ Lack of laboratory infrastructure
- ▢ Lack of access to latest techniques and technologies
- ▢ Lack of good manufacturing practices
- ▢ Lack of access to online databases and software
- ▢ Lack of time for you as a researcher
- ▢ Lack of trained personnel/training
- ▢ Other, please specify ____
- ▢ I do not perceive any barriers

Q16 Based on your response to question 15, which of the ones you selected is the most important barrier(s)? Please comment in the box below.

____

Q17 What would help you to overcome the existing barriers in medicinal plant extract characterisation (e.g. pharmacological, toxicological and/or clinical/intervention studies)? Please select all that apply.

- ☐ Rigorous and sustainable supply chain of plant material/herbal substances
- ☐ Published mandates and guidelines by academic journals
- ☐ Platforms for accessing specific collaborative links
- ☐ Better equipped laboratory facilities
- ☐ Accessible online databases and software
- ☐ Other, please specify ____
- ☐ Nothing

Q18 What do you think could be improved in pharmacological, toxicological and/or clinical/intervention studies using medicinal plant extracts? Please comment in the box below.

____

Q19 What do you think needs to be improved in medicinal plants research more generally?

____

Other comments/suggestions, please write them down in here

____

____

____

____

____

End of Block: Challenges and Barriers for medicinal plant extract characterisation

---

Start of Block: Thank you very much for completing this survey.
