# Supplementary material for: Best Practice in the chemical characterisation of extracts used in pharmacological and toxicological research—The ConPhyMP—Guidelines
Source: Front Pharmacol. 2022 Sep 13;13:953205. doi: 10.3389/fphar.2022.953205 (PMC9514875; doi:10.3389/fphar.2022.953205)
Supplement: Supplementary file 3 [file Table2.pdf]

TABLE S2 Characteristics of the survey respondents (n=363).

**Characteristics**	**Frequency (%)**
**Gender**	
Male	204 (56.2%)
Female	153 (42.1%)
Non-binary/third gender	4 (1.1%)
Prefer not to say	2 (0.6%)
**Age (years)**	
21-30	52 (14.3%)
31-40	109 (30%)
41-50	85 (23.4%)
51-60	64 (17.6%)
61-70	34 (9.4%)
Over 71	19 (5.2%)
**Type of organisation**	
Academia/University	266 (73.3%)
Government	21 (5.8%)
Independent Research Centre	9 (2.5%)
Industry	41 (11.3%)
Other	21 (5.8%)
Prefer not to say	5 (1.4%)
**Career level**	
PhD student/researcher	81 (22.3%)
Postdoctoral researcher	39 (10.7%)
Lecturer	24 (6.6%)
Senior Lecturer/Reader	36 (9.9%)
Professor	107 (29.5%)
Other	68 (18.7%)
Prefer not to say	8 (2.2%)
**The position they held. Respondent could chose more than one answer). Dichotomy group tabulated at value 1** (n=363; multiple responses possible)	
Director/Head of Institute/Department	60 (16.5%)
Editor In Chief/Associate Editor	42 (11.6%)
Director/Head of Research Centre	16 (4.4%)
Head of Research group	88 (24.2%)
Manager/Supervisor	36 (9.9%)
Consultant	28 (7.7%)
Researcher	174 (47.9%)
Other	32 (8.8%)

I do not hold any position	34 (9.4%)
Prefer not to say	3 (0.8%)
**Country/Continent they are based in[1]**	
Africa - Northern Africa	9 (2.5%)
Africa - Sub-Saharan Africa	41 (11.3%)
Americas - Latin America and the Caribbean	22 (6.1%)
Americas - Northern America	38 (10.5%)
Asia - Central Asia	9 (2.5%)
Asia - Eastern Asia	39 (10.7%)
Asia - South-eastern Asia	42 (11.6%)
Asia - Southern Asia	44 (12.1%)
Asia – Western Asia	7 (1.9%)
Europe - Eastern Europe (including Northern Asia)	15 (4.1%)
Europe - Western Europe (including the Mediterranean and Northern Europe)	91 (25.1%)
Oceania - Australia and New Zealand	6 (1.7%)

[1]The division of the countries and territories of the world into regional and subregional groups used is based on the United Nations geoscheme system. It was devised by the United Nations Statistics Division (UNSD) based on the M49 coding classification.
