# Supplementary material for: Best Practice in the chemical characterisation of extracts used in pharmacological and toxicological research—The ConPhyMP—Guidelines
Source: Front Pharmacol. 2022 Sep 13;13:953205. doi: 10.3389/fphar.2022.953205 (PMC9514875; doi:10.3389/fphar.2022.953205)
Supplement: Supplementary file 4 [file Table3.pdf]

TABLE S3 Main focus of research concerning medicinal plants (n=328; multiple responses possible).

Main focus of research. Respondent can chose more than one answer). Dichotomy group tabulated at value 1	Frequency	% of Cases
Anti-inflammatory activity	147	44.82%
Antioxidant activity	140	42.68%
Antimicrobial activity	115	35.06%
Cytotoxicity activity	104	31.71%
Anti-cancer activity	99	30.18%
The gastrointestinal and biliary system	77	23.48%
The central nervous system	71	21.65%
Infectious diseases	68	20.73%
Anti-ageing	55	16.77%
Other	53	16.16%
The cardiovascular system	48	14.63%
The skin	47	14.33%
The endocrine system	41	12.50%
The respiratory system	41	12.50%
Cosmetics	39	11.89%
Stress management	36	10.98%
Weight loss	28	8.54%
The reproductive tract	20	6.10%
Psychoactive	15	4.57%
The musculoskeletal system	15	4.57%
Aphrodisiac	13	3.96%
The eye	7	2.13%
Hallucinogen	5	1.52%
Ear, nose and oropharynx	5	1.52%
